# A leave-one-out algorithm for contribution analysis in component network meta-analysis

**DOI:** 10.1186/s12874-025-02619-w

**Published:** 2025-08-07

**Authors:** Yunhe Mao, Yiwen Shen, Qinbo Yang, Qingyang Shi, Sheyu Li

**Affiliations:** 1 Sports Medicine Center, West China Hospital, Sichuan University, Chengdu, China; 2 Department of Orthopedics and Orthopedic Research Institute, West China Hospital, Sichuan University, Chengdu, China; 3 School of Business and Management, The Hong Kong University of Science and Technology, Hong Kong, Hong Kong SAR China; 4 Department of Nephrology, West China Hospital, Sichuan University, Chengdu, China; 5 Groningen Research Institute of Pharmacy, Faculty of Science and Engineering, University of Groningen, Groningen, Netherlands; 6 Department of Endocrinology and Metabolism, MAGIC China Center, West China Hospital of Sichuan University, No.37 Guoxue Alley, Wuhou District, Chengdu City, Sichuan Province China

**Keywords:** Component network meta-analysis, Contribution, Evidence synthesis

## Abstract

**Background:**

Component network meta-analysis (CNMA) enables disentangling individual component effects from multicomponent treatments. However, no established methods exist to quantify the contribution of evidence from constituent comparisons to the disentangled component effect estimates in CNMA, hindering the interpretability of results.

**Methods:**

We proposed a leave-one-out algorithm to address this gap. The core approach iteratively excludes each constituent comparison (i.e., edge in the network), recomputes the variances of all component effects, and quantifies the precision leverage of each comparison based on the induced variance inflation. Contributions are assigned via a normalized matrix. We developed special rules to handle cases where exclusion renders component effects unidentifiable. The method also formally decomposes component estimates into direct and additive evidence sources. Its utility and validity were evaluated through implementation using hypothetical networks and a real-world dataset.

**Results:**

The leave-one-out algorithm accurately identified pivotal evidence sources by capturing substantial variance fluctuations upon their exclusion. Contributions assigned via precision leverage effectively quantified the critical importance of comparisons isolating target components. Application to real-world data (66 comparisons, 21 components) also confirmed the method’s precision in discerning influential evidence within complex networks, and exhibited strong alignment with the parameter decomposition results. Crucially, validation revealed no inherent relationship exists between precision leverage and linear weighting.

**Conclusions:**

The leave-one-out algorithm resolves a critical gap in CNMA methodology by providing a robust, variance-based framework for quantifying the contribution of constituent direct comparisons to component effect estimates. It reliably identifies pivotal evidence sources essential for component identifiability and precision across diverse network structures, enhancing the transparency and interpretability of evidence synthesis for complex interventions.

## Introduction

Network meta-analysis (NMA) is an advanced statistical methodology that extends pairwise meta-analysis principles [1, 2]. It enables simultaneous comparisons of three or more interventions for a medical condition through the integration of both direct evidence from clinical trials and indirect evidence from connected treatment networks [3, 4]. NMA operates at the treatment-as-a-whole level, synthesizing evidence through predefined treatment nodes that represent complete therapeutic regimens [5]. This node-based approach, however, faces inherent limitations when analyzing multi-component interventions. These interventions, such as cognitive behavioral therapy regimens, typically integrate multiple active components [6–8]. NMA falls short in disentangling the individual effects of each component within complex interventions. This limitation hampers the utility of NMA for guideline developers who require specific recommendations on intervention ingredients or modules [9, 10].

Component network meta-analysis (CNMA) addresses this gap through a paradigm shift - rather than analyzing interventions as monolithic entities, it systematically decomposes them into constituent components (e.g., behavioral modules) to quantify their additive and interactive effects, where the overall effect equals the sum of individual component estimates plus their potential interactions [11–14]. This mathematical formalization enables precise estimation of both the incremental value attributable to each discrete component and synergistic/antagonistic effects emerging from component combinations. This analytical approach empowers guideline developers to address critical questions such as, *“Which components contribute most significantly to efficacy?”* or *“Can we optimize interventions by adding or removing modules?".*

While CNMA holds significant potential for advancing evidence-based intervention design, its evidence interpretation hinges on establishing a robust analytical framework. Such a framework must precisely delineate how pairwise comparisons contribute to component-level estimations through mathematical decomposition of treatment nodes, and systematically quantify the propagation of study-level information across the entire evidence network.

In traditional NMA, evidence contribution quantification relies on graph-theoretic path tracing, such as ‘random walk’ or ‘shortest path’, that maps evidence flows through observable treatment comparisons (i.e., edges) [15–18]. However, CNMA introduces a hierarchical structure: components are nested within treatments, and treatment effects are linear combinations of component effects. This structural shift renders traditional contribution analyses inadequate due to the unidentifiability of component pathways and combinatorial complexity. Overlapping components across treatments create interdependencies in evidence flow, precluding the isolation of individual component contributions through pathway enumeration. Furthermore, while traditional NMA relies on direct comparisons from observed data, CNMA introduces latent connections between the treatments having common components—comparisons inferred under the additivity assumption rather than observed in trials [14]. For instance, a comparison between “A + B” and “A + C” implicitly informs the effect difference between components B and C, even without direct trials. Consequently, even if pathway-specific contributions could theoretically be calculated, the integration of hypothetical edges undermines the ability to evaluate evidence quality along composite pathways, as these paths may conflate empirical and model-derived comparisons.

To address these limitations, we proposed a leave-one-out algorithm that evaluates the influence of each constituent direct comparison on the statistical precision of component effect estimates, which is grounded in jackknife principles and influence diagnostic theory [19–21]. This leave-one-out concept has been suggested in many statistical contexts, Rücker had proposed similar leave-one-out methods earlier for contribution analyses in treatment-level NMA [22]. However, this study is the first to define proportional contributions of direct estimates to component estimates in CNMA. Below, we provide rigorous mathematical formulations, algorithmic steps, and illustrative examples to delineate the theoretical and computational foundations of this method.

## Methods

### Conceptualizing contribution in CNMA

In this study, we define *contribution* as a statistical leverage metric quantifying the unique influence of each comparison on the precision of component effect estimates, analogous to Cook’s distance in regression diagnostics [19]. This approach employs an iterative leave-one-out procedure: for each comparison, we recompute component variances after its exclusion from the network. This isolates the comparison’s unique impact on variance inflation, measuring how it stabilizes the precision of component estimates. Unlike more recent pathway-based decomposition methods that quantify contribution via evidence flow metrics, this precision-centric metric directly links comparisons to parameter identifiability through variance modulation [15–17].

All analyses presented in this study are based on the common effect model (traditionally termed the ‘fixed effect model’). This model assumes a single true effect size underlying each comparison, primarily for conceptual clarity and to facilitate the methodological exposition.

### Key parameters

In the CNMA, treatment effects are mathematically decomposed into linear combinations of their constituent components. Consider a set of $\:n$ distinct components $\:\mathcal{C}=\{{c}_{1},{c}_{2},\dots\:,{c}_{n}\}$(e.g., specific drug ingredients or behavioral modules) and $\:y$ treatments $\:\mathcal{T}=\{{T}_{1},{T}_{2},\dots\:,{T}_{y}\}$, where each treatment $\:{T}_{i}\subseteq\:\mathcal{C}$ is a subset of components. In the additive model, the core assumption is that the effect of a multicomponent treatment equals the sum of its individual components’ effects, expressed as $\:{\beta\:}_{T}={\sum\:}_{{c}_{j}\in\:T}{\beta\:}_{{c}_{j}}$ for treatment $\:T$. Consequently, when comparing two treatments, shared components cancel out, and the observed contrast reflects only the effect of non-overlapping components. The additive model estimates the effects of individual components $\:\:\widehat{\beta\:}\:\in\:{R}^{n}$ using weighted least squares [23], expressed as:

$$\:\widehat{\beta\:}={\left({X}_{a}^{\top\:}{WX}_{a}\right)}^{+}{X}_{a}^{\top\:}Wd$$

The variances of the component effects are derived from the diagonal elements of covariance matrix of $\:{\left({X}_{a}^{\top\:}{WX}_{a}\right)}^{+}$. The variance for component $\:{c}_{j}$ is given by:

$$\:{\text{V}}_{\text{j}}={\left[{\left({X}_{a}^{\top}W{X}_{a}\right)}^{+}\right]}_{jj}$$

### Leave-one-out method

In this approach, we operate at the level of comparisons (edges in the network), not individual studies. Each direct comparison $\:{e}_{i}$ represents a contrast between interventions and may be supported by one or multiple studies. The contribution of each $\:{e}_{i}$ to the estimation of a component effect $\:{\beta\:}_{j}$ is quantified by its “precision leverage”—the relative increase in the variance of $\:{\beta\:}_{j}$ when $\:{e}_{i}$ is excluded from the network. This generalizes Rücker et al.’s “importance” metric for treatment-level NMA estimates to component-level inference [22]: `.

The leave-one-out algorithm proceeds as follows:

1. Full network estimation: conduct a CNMA for the given network to estimate all component effects ${\beta\:}_{{full,\: j}}$ and their variances ${V}_{{full,\:j}}$ using the `netcomb` or `discomb` functions from the *netmeta* package (version 3.2-0).
2. Leave-one-out iteration: for each direct comparison $\:{e}_{i}$, conduct a CNMA without $\:{e}_{i}$ and recompute all component effects $\:{\beta\:}_{-i,j}$ and their variances $\:{V}_{-i,j}$ iteratively.
3. Precision leverage calculation: define the precision leverage of $\:{e}_{i}$ for $\:{\beta\:}_{j}$ as:

1 $${m}_{i,j}=\frac{{V}_{-i,\:j}-{{V}}_{{full},\:{j}}}{{V}_{-i,\:j}}=1-\frac{{{V}}_{{full},\:{j}}}{{V}_{-i,\:j}}$$

We have 0 ≤ $\:{m}_{i,j}$ ≤ 1 for all direct comparison $\:{e}_{i}$ and component $\:{c}_{j}$. The interpretation of metric $\:m$ here is, if exclusion of $\:{e}_{i}$ causes no variance increase (i.e., $\:{m}_{i,j}=0$), then $\:{e}_{i}$ is redundant for estimating $\:{\beta\:}_{j}$; if exclusion of $\:{e}_{i}$ maximally increases variance (i.e., $\:{m}_{i,j}=1$), then $\:{e}_{i}$ is absolutely essential for estimating $\:{\beta\:}_{j}$. Higher values of $\:m$ indicate greater leverage on the precision (which is more important).

However, there are special scenarios that require additional methodological considerations. Specifically, if the exclusion of a comparison $\:{e}_{i}$ causes $\:{c}_{j}\:$to be unidentifiable, the contributions are redistributed across conflicting contrasts based on certain rules. Detailed descriptions of these rules are provided in the Method section of *“*Redistribution of contribution under unidentifiability”, and their implementations are in the Results section of “Hypothetical networks”.

### Redistribution of contribution under unidentifiability

During the process of iteratively leaving each comparison out of a given network, especially in sparse networks, we found that the deletion of certain comparisons would render $\:{\beta\:}_{-i,j}$ unidentifiable (i.e., $\:{V}_{-i,j}\to\:{\infty\:}$), the standard formula of $\:{m}_{i,j}$ becomes undefined, necessitating alternative rules for contribution assignment.

#### Scenario 1: single comparison causing unidentifiability

If the exclusion of a specific comparison $\:{e}_{x}$ results in the unidentifiability of target component $\:{c}_{j}$, then $\:{e}_{x}$ is deemed “absolutely essential” for estimating the component. Here, the precision leverage is assigned as:

$$\:{m}_{x,j}=1.$$

This assignment reflects that $\:{e}_{x}$ provides unique information critical to the identifiability of $\:{c}_{j}$, without which the component effect $\:{\beta\:}_{j}\:$cannot be estimated. In other words, the exclusion of $\:{e}_{x}$ induces infinite variance inflation for $\:{\beta\:}_{j}$. This occurs because $\:{e}_{x}$ is the sole conduit for evidence about $\:{c}_{j}$ in the network, its removal collapses all estimation pathways for the component.

#### Scenario 2: multiple comparisons causing unidentifiability

When a set of comparisons $\:{\mathcal{E}}_{\mathcal{j}}=\{{e}_{1},{e}_{2},\dots\:,{e}_{n}\}$ exhibits the property that the exclusion of any single member $\:{e}_{k}\in\:{\mathcal{E}}_{\mathcal{j}}$ causes unidentifiability of $\:{\beta\:}_{j}$, these comparisons collectively form a “minimal identifying set” for the component. In such cases, the total identifiability contribution of $\:{\mathcal{E}}_{\mathcal{j}}$ is uniformly distributed among its members. For each comparison $\:{e}_{k}\in\:{\mathcal{E}}_{\mathcal{j}}$, the precision leverage is assigned as:

$$\:m_{k,j}=\frac1n,\quad n=\left|{\mathcal{E}}_{\mathcal{j}}\right|.$$

The rationale of this assignment is that each $\:{e}_{k}\in\:{\mathcal{E}}_{\mathcal{j}}$ is necessary for the identifiability of $\:{\beta\:}_{j}$, as its removal alone induces rank deficiency in the “information matrix” $\:{X}_{a}^{\top\:}{WX}_{a}$. However, no single $\:{e}_{k}$ is sufficient to ensure identifiability; the entire set $\:{\mathcal{E}}_{\mathcal{j}}$ is required for a well-defined estimate. The set $\:{\mathcal{E}}_{\mathcal{j}}$ is minimal in the sense that (i) no proper subset of $\:{\mathcal{E}}_{\mathcal{j}}$ guarantees identifiability, and (ii) the removal of any $\:{e}_{k}\in\:{\mathcal{E}}_{\mathcal{j}}$ disrupts all paths that isolate $\:{\beta\:}_{j}$ in the network.

### Contribution matrix

To quantify and compare the relative influence of each direct comparison $\:{e}_{i}$ on the precision of each component effect $\:{\beta\:}_{j}$, we introduce a normalized percentage contribution matrix $\:M\in\:{R}^{n\times\:k}$, where $\:n$ is the number of distinct components and $\:k$ is the number of edges. Each element $\:{M}_{i,j}$ represents the percentage contribution of comparison $\:{e}_{i}$ to the precision of the estimate for component $\:{c}_{j}$.

This normalization addresses a key limitation of the raw leverage values $\:{m}_{i,j}$ (Eq. 1). While $\:{m}_{i,j}$ quantifies the absolute change when excluding $\:{e}_{i}$, it doesn’t reveal how important it is relative to other comparisons $\:{e}_{i{\prime\:}\ne\:i}$. Therefore, to enable direct comparison and interpretation of the relative contribution weights across comparisons for each component $\:{c}_{j}$, we normalize the leverage values $\:{m}_{i,j}$ to sum to 100% per component:

2 $$\:{M}_{i,j}=\frac{{m}_{i,j}}{{\sum\:}_{{i}^{{\prime\:}}=1}^{k}{m}_{{i}^{{\prime\:}},j}}\times\:100\%$$

### Decomposition of component estimate

In the CNMA, we use additive evidence instead of indirect evidence in NMA to differentiate the methodology scenarios. To rigorously formalize the relationship between direct and additive evidence proportions, we derive the decomposition process by extending Rücker et al.’s variance-based importance algorithm in NMA to additive CNMA [22]:

If direct comparisons exist for standalone component $\:{c}_{j}$ (e.g., $\:{c}_{j}$ vs. control), the direct effect $\:{\beta\:}_{\text{dir},j}$ is obtained via pairwise meta-analysis (as we conduct all analyses at edge level). We denote the variance estimates of the component effect estimate (also called incremental effect in *netmeta* package), the direct effect estimate and the additive effect estimate of target component $\:{c}_{j}$ by $\:{V}_{\text{com},j}$, $\:{V}_{\text{dir},j}$, $\:{V}_{\text{add},j}$ and the inverse variance weights by $\:{w}_{\text{com},j}={{(V}_{\text{com},j})}^{-1}$,…and so on. Under the assumption that direct and additive evidence is independent, the inverse-variance relationship holds:

3 $$\:{{(V}_{\text{com},j})}^{-1}={{(V}_{\text{dir},j})}^{-1}+{{(V}_{\text{add},j})}^{-1}=\frac{{V}_{\text{dir},j}+{V}_{\text{add},j}}{{V}_{\text{dir},j}\times\:{V}_{\text{add},j}}$$

rearranging, we compute:

4 $$\:\frac{{V}_{\text{com},j}}{{V}_{\text{add},j}}=\frac{{V}_{\text{dir},j}-{V}_{\text{com},j}}{{V}_{\text{dir},j}}=1-\frac{{V}_{\text{com},j}}{{V}_{\text{dir},j}}$$

5 $$\:\frac{{V}_{\text{com},j}}{{V}_{\text{dir},j}}=1-\frac{{V}_{\text{com},j}}{{V}_{\text{add},j}}=\frac{{V}_{\text{add},j}-{V}_{\text{com},j}}{{V}_{\text{add},j}}$$

and in terms of inverse variance weights:

6 $$\:\frac{{V}_{\text{com},j}}{{V}_{\text{dir},j}}=1-\frac{{w}_{\text{add},j}}{{w}_{\text{com},j}}=\frac{{w}_{\text{com},j}-{w}_{\text{add},j}}{{w}_{\text{com},j}}$$

The direct evidence proportion $\:{\varphi\:}_{\text{dir},j}$ of the incremental effect of component $\:{c}_{j}$ can be defined through the inverse variance weights as:

7 $$\:{\varphi\:}_{\text{dir},j}=\frac{{w}_{dir,j}}{{w}_{\text{dir},j}+{w}_{\text{add},j}}=\frac{{V}_{add,j}}{{V}_{\text{dir},j}+{V}_{\text{add},j}}$$

And we obtain:

8 $$\:{\varphi\:}_{\text{dir},j}=\frac{{V}_{\text{com},j}}{{V}_{\text{dir},j}};\hspace{1em}1-{\varphi\:}_{\text{dir},j}=\frac{{V}_{\text{com},j}}{{V}_{\text{add},j}}={\varphi\:}_{\text{add},j}$$

This decomposition formalizes how direct and additive evidence contribute to component effect estimates in CNMA under the independence assumption. The direct evidence proportion $\:{\varphi\:}_{\text{dir},j}=\frac{{V}_{\text{com},j}}{{V}_{\text{dir},j}}$ represents the relative size of the component variance compared to the direct estimate alone—e.g., $\:{\varphi\:}_{\text{dir},j}=0.3$ indicates $\:{V}_{\text{com},j}$ is 30% of $\:{V}_{\text{dir},j}$. Conversely, the additive evidence proportion $\:\:{\varphi\:}_{add,j}\:=\frac{{V}_{\text{com},j}}{{V}_{\text{add},j}}$ quantifies the precision share of additive evidence in the component estimate. Under independence, this proportion also equals the relative variance reduction achieved when augmenting direct evidence with additive evidence (Eq. 4, $\:{\varphi\:}_{add,j}=\frac{{V}_{\text{com},j}}{{V}_{\text{add},j}}=1-\frac{{V}_{\text{com},j}}{{V}_{\text{dir},j}}$). Thus, $\:{\varphi\:}_{add,j}$ has dual interpretations:

1. As a precision share, it reflects additive evidence’s contribution to reducing overall uncertainty.
2. As a variance reduction metric, it measures efficiency gain from evidence integration.

Each evidence stream’s contribution to the composite precision is proportional to its inverse variance (Eq. 7), extending Rücker’s framework to CNMA [22].

### Real-world dataset implementation and validation

#### Dataset

We utilized original data from our research team’s investigation into the effects of exercise modalities on lean mass proportion in adults with obesity. This comprehensive dataset comprises 66 treatment comparisons across 21 components (distinct exercise intervention modalities), providing a robust foundation for empirical implementation of the leave-one-out contribution analysis framework.

#### Validation methodology

We established an internal validation protocol using linear weighting to generate surrogate ground truth values. For each component $\:{c}_{j}$, the predicted effect size $\:{\beta\:}_{\text{pred},j}$ was derived through the following linear combination:

$$\:{\beta\:}_{\text{pred},j}={\sum\:}_{i=1}^{k}{M}_{i,j}\cdot\:{d}_{i}$$

Where $\:{M}_{i,j}$ is the normalized contribution percentage of comparison $\:{e}_{i}$ to component $\:{c}_{j}$ (from contribution matrix); $\:{d}_{i}$is the observed effect size of direct comparison $\:{e}_{i}$ from pairwise meta-analysis; $\:k$ is the number of comparisons. The summation was performed across all treatment comparisons. We evaluated congruence between predicted effects $\:{\beta\:}_{\text{pred},j}$ and additive model component estimates $\:{\beta\:}_{j}$ using three complementary metrics:

i. Pearson correlation ($\:\text{r}$): measures linear association
ii. Explained variance ($\:{\text{R}}^{2}$): quantifies proportion of variance captured
iii. Mean absolute error (MAE): $\:\text{MAE}=\frac{1}{\text{n}}{\sum\:}_{\text{j}=1}^{\text{n}}\left|{\beta\:}_{\text{com},j}-{\beta\:}_{\text{pred},j}\right|$

Statistical significance of correlation was evaluated through Pearson’s test with Holm-adjusted p-values. Residual distributions were characterized by:

i. Absolute difference thresholds: Low (< 0.16), Medium (0.16–0.24), High (> 0.24).
ii. Percentile analysis: 67% of residuals below the 66th percentile (0.24 units).

#### Rationale for validation

In standard NMA, contribution weights derived from pathway-based enumeration strictly satisfy $\:{\beta\:}_{\text{network}}=\sum\:{M}_{i,j}\cdot\:{d}_{i}$ by design [23], as they represent explicit evidence flow. However, it is highly improbable for this equivalence to hold in the CNMA due to fundamental methodological divergence. Our linear weighting generates $\:{\beta\:}_{\text{pred},j}$ not as an expected equivalent to $\:{\beta\:}_{\text{com},j}$, but as a diagnostic tool to quantify the divergence between precision-based leverage and pathway-based evidence flow contributions. The congruence metrics thus measure conceptual alignment—not mathematical equivalence—between these distinct dimensions of “contribution”.

### Premise assumptions

The leave-one-out algorithm relies on two foundational assumptions to ensure methodological validity in CNMA. Violations of these assumptions may compromise the reliability of component effect estimates.

1. Linear additivity

The observed treatment effect for any intervention

$\:T\subset\:\mathcal{C}$ is the algebraic sum of its constituent component effects:

$$\:{\beta\:}_{T}={\sum\:}_{{c}_{j}\in\:T}{\beta\:}_{j}$$

This mirrors additivity models where combination therapies exhibit no interactions. Violations (e.g., synergistic or antagonistic effects) invalidate the additive structure and require extension to interaction models. To examine this crucial additivity assumption, Rücker et al. propose a test of additivity which is based on the comparison of treatment estimates from the standard NMA and the additive model in CNMA [14].

Specifically, the test evaluates the discrepancy between the two models using heterogeneity statistics. The NMA model estimates treatment effects $\:{\widehat{{\updelta\:}}}^{\text{nma}}=Hd$, where $\:H$ is the hat matrix projecting observed effects $\:d$ onto a subspace of consistent contrasts. The heterogeneity statistic $\:Q={\left(d-{\widehat{\delta\:}}^{\text{nma}}\right)}^{{\top\:}}W\left(d-{\widehat{\delta\:}}^{\text{nma}}\right)$ follows a chi-square distribution with $\:df={n}_{a}-k-\left(n-1\right)$ degrees of freedom.

The additive model assumes linear additivity, estimating effects$\:\:{\widehat{{\updelta\:}}}_{a}={H}_{a}d$, where $\:{H}_{a}$ is the projection matrix for the additive structure. The corresponding heterogeneity statistic $\:{Q}_{a}={\left(d-{\widehat{\delta\:}}_{a}\right)}^{\top\:}W\left(d-{\widehat{\delta\:}}_{a}\right)\:$has $\:d{f}_{a}={n}_{a}-k-r$ degrees of freedom, with $\:r$ being the rank of the $\:{X}_{a}$.

The test statistic$\:\:{Q}_{a}-Q={\left({\widehat{\delta\:}}_{a}-{\widehat{\delta\:}}^{\text{nma}}\right)}^{\top\:}W\left({\widehat{\delta\:}}_{a}-{\widehat{\delta\:}}^{\text{nma}}\right)$, distributed as $\:{{\upchi\:}}^{2}$with $\:{\Delta\:}df\:=\:n\:-\:r\:-\:1$, quantifies the divergence between models. A nonsignificant result $\:p\ge\:0.05$ supports additivity, indicating no substantial unmodeled interactions. Conversely, significance $\:p<0.05$ rejects additivity, necessitating interaction terms or flagging out problematic components.

(2) Identifiability of component in full network

Reliable estimation of component effects depends on their independent identifiability through additive relationships in the full evidence network. This requires that the design matrix $\:{\varvec{X}}_{\varvec{a}}$ encode sufficient contrasts to disentangle component effects. If components are linearly dependent (e.g.,$\:{c}_{1}$ and $\:{c}_{2}$ always co-administered), their effects cannot be disentangled without additional constraints (e.g., fixing one effect to certain value) [24, 25].

## Results

We first apply the leave-one-out method to a number of hypothetical examples that nevertheless lead to insight into the interpretation of the measure of contribution. Then we demonstrate its utility in a real-world dataset.

### Hypothetical networks

#### Network 1

**Fig. 1:**
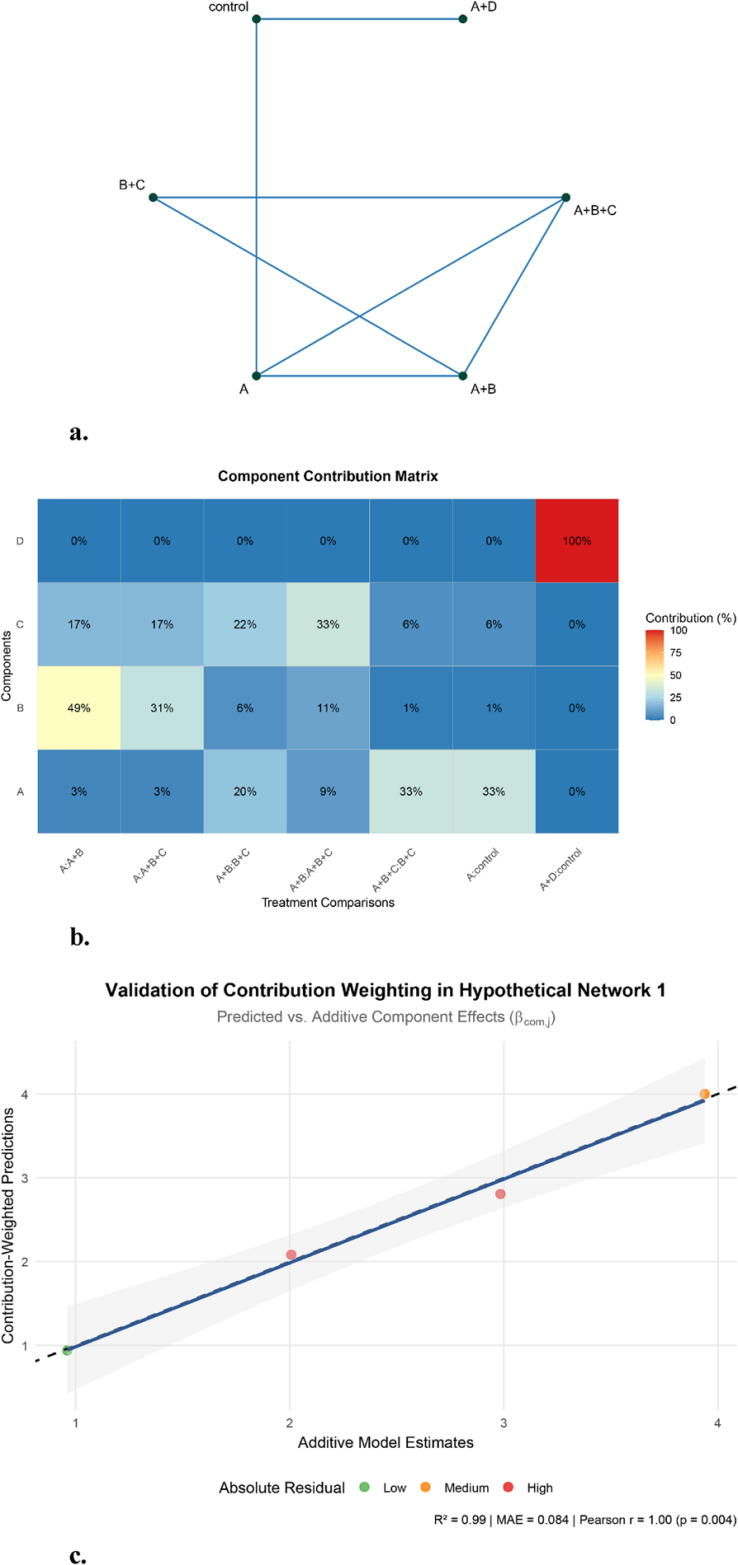
**a** Network plot for hypothetical network 1. For simplicity in understanding precision leverage variations, the variance of all comparisons in this hypothetical network was set to 1. **b** Contribution matrix using leave one out method. This example is to demonstrate contribution distribution under equal variance of each comparison, and to show contribution assignment under single comparison causing unidentifiability (component D). **c** Validation of contribution-weighted predictions in hypothetical network 1. Scatterplot shows congruence between additive model estimates ($\:{\beta\:}_{\text{com}}$) and contribution-weighted predictions ($\:{\beta\:}_{\text{pred}}$). Dashed line = identity reference; blue band = 95% CI of regression slope. Key metrics: (1) R² (0.99): variance explained; (2) MAE (0.084): average prediction error; (3) Pearson r (1.00): linear correlation strength. Residual colors denote absolute difference tertiles: low (≤ 0.12, green), medium (0.12–0.24, orange), high (> 0.24, red)

The structure of the hypothetical network 1 is shown in Fig. 1a, and Fig. 1b displays the contribution proportion matrix derived using our leave-one-out method. For simplicity in understanding precision leverage variations, the variance of all comparisons in this hypothetical network was set to 1. A crucial perspective when analyzing the effects of comparisons in CNMA involves canceling out common components on both sides of a comparison to reveal the actual estimated effect.

For component A, the comparisons `A vs. control` and `A + B + C vs. B + C` each account for 33% of the contribution proportion. Notably, `A + B + C vs. B + C` essentially estimates the effect of `A vs. control`, and they contributed exactly the same. These two comparisons play the primary role in determining the component effect of A. Conversely, the comparisons `A vs. A + B` and `A vs. A + B + C` each contribute only about 2% to the effect of A. The reason is that after canceling out common components (these comparisons become equivalent to `control vs. B` and `control vs. B + C`), they do not directly involve the estimation of A itself, rendering them ‘less important’ for determining A’s component effect. The comparisons “A + B vs. B + C” and “A + B vs. A + B + C” collectively contribute 29% because they jointly constrain component C. Since component A appears in comparisons with C (“A + B vs. B + C” = A vs. C), uncertainty in C propagates to A. By providing complementary information on C—one directly (control vs. C) and one relatively (A vs. C)—these comparisons reduce the effective correlation between A and C in the model. This collinearity reduction sharpens the estimate of A, even though neither comparison isolates A alone.

Similarly, for component B, the comparison `A vs. A + B` (49%) plays a decisive role, as it essentially compares `B vs. control`. It is noteworthy that the comparison `A vs. A + B + C` (31%) also contributes substantially. A plausible explanation is that the presence of B within the effect estimate of B + C (which isn’t isolated in this comparison) is important for determining B’s effect. The remaining comparisons, which do not directly involve B’s isolated effect, contribute minimally (e.g., `A + B vs. B + C` (6%), `A + B vs. A + B + C` (11%), `A vs. control` and `A + B + C vs. B + C` (each 1%)).

The contribution distribution for component C is less typical but still interpretable. The comparison `A + B vs. A + B + C` (33%) plays a decisive role, as it directly estimates `C vs. control`. Other comparisons containing C, namely `A vs. A + B + C` (17%) and `A + B vs. B + C` (22%), also contribute significantly.

For component D, only the comparison `A + D vs. control` contains its information. Critically, removing `A + D vs. control` renders D unidentifiable (its information is completely lost). This corresponds to ***Scenario 1*** described in the section “Re-distribution of contribution under unidentifiability”. Consequently, the contribution of `A + D vs. control` to D is assigned as 100%. Crucially, because this exclusion makes D’s variance infinite, all other comparisons—regardless of their individual removal—can no longer provide meaningful leverage on D’s precision. Their calculated precision leverages become incomparable under infinite variance, leading the algorithm to assign them 0% contribution for component D.

Figure 1c shows the congruence test between the predicted component estimate and the model derived component estimate. High congruence was validated between the contribution-weighted component effects $\:{\beta\:}_{\text{pred}}$ and the model derived $\:{\beta\:}_{\text{com}}$ for these four components (R²=0.99; MAE = 0.084; Pearson *r* = 1.00). However, it is crucial to interpret this alignment with caution: this observed consistency is largely an artifact of the simplified structure and the artificial setting where all comparisons share identical variance (set to 1). The contribution proportions $\:{M}_{i,j}$fundamentally measure the statistical leverage of a comparison on the *precision* of the component estimate, not its direct role as a weight for determining the effect size ($\:{\beta\:}_{j}$) in the additive model. Therefore, this numerical agreement in the hypothetical example should not be misconstrued as validating the use of $\:{M}_{i,j}$ for linear weighting to estimate component effects; they represent distinct concepts, and as the subsequent real-world dataset validation will demonstrate, such strong agreement does not hold in more complex and realistic settings with heterogeneous variances.

#### Network 2

**Fig. 2:**
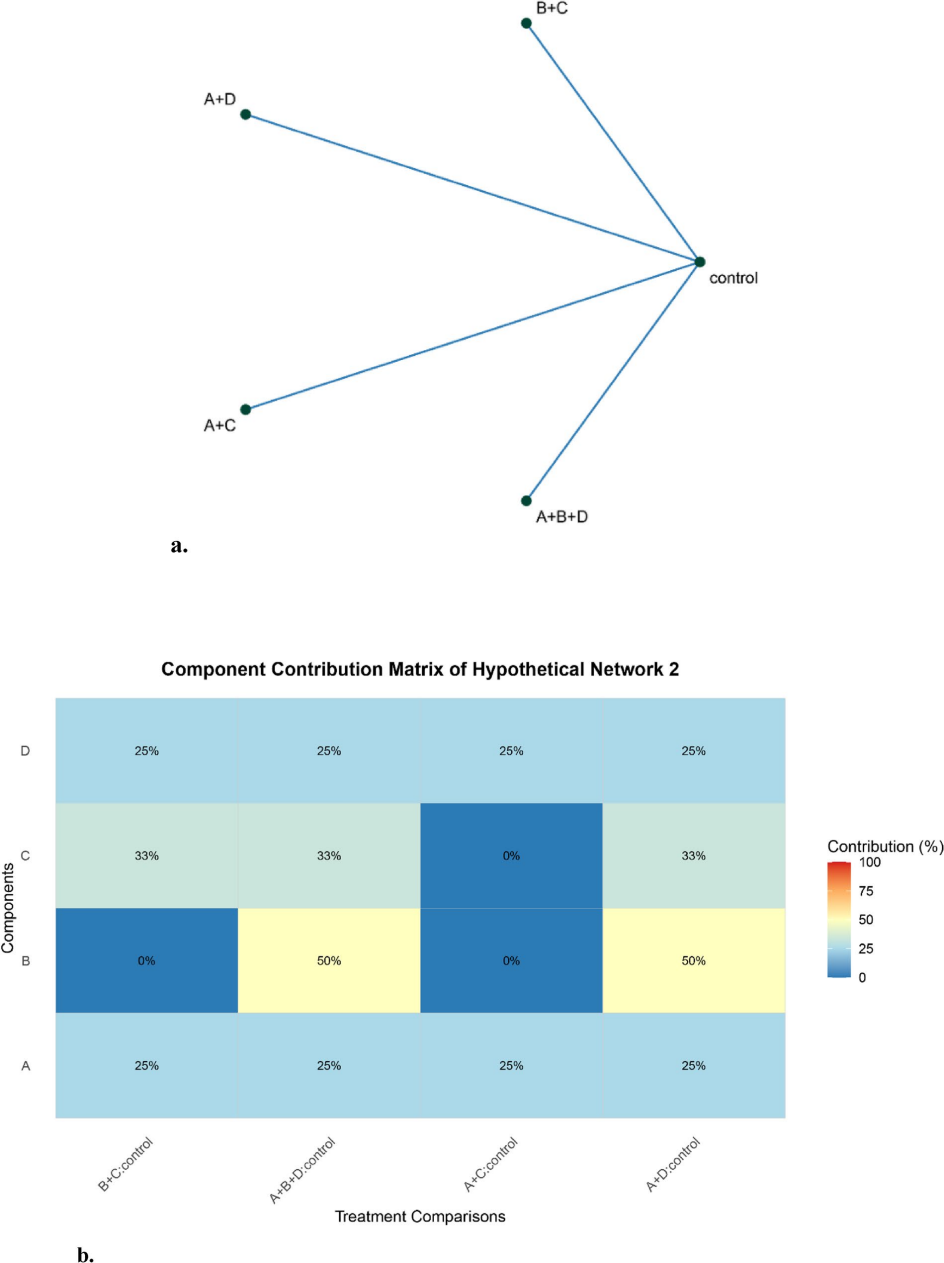
**a** Network plot for hypothetical network 2. This example is to show the scenario that removing any single comparison in a “minimal identifying set” would disrupt identifiability for certain components. **b** Contribution matrix using redistribution rules under unidentifiability. This example is to demonstrate contribution redistribution under multiple comparisons causing unidentifiability

Figure 2a presents a specific network example involving comparisons including `A + B + D vs. control`, `B + C vs. control`, `A + C vs. control`, and `A + D vs. control`, all components (A, B, C, D) are initially identifiable in the full network. However, removing any single comparison disrupts identifiability for certain components. These comparisons comprise the typical “minimal identifying set” mentioned in the contribution redistribution ***Scenario 2***.

For component A, deleting any one of the four comparisons renders A unidentifiable (flagged by the system), indicating that all four comparisons collectively form a minimal identifying set for A, with each contributing a precision leverage of 25%.

Similarly, component D becomes unidentifiable if any comparison is removed, leading to the same uniform contribution of 25% from each comparison.

For component B, only the removal of `A + B + D vs. control` or `A + D vs. control` causes unidentifiability, resulting in both comparisons equally contributing 50% to B’s identifiability.

Component C, on the other hand, loses identifiability when any of three comparisons—`A + B + D vs. control`, `B + C vs. control`, or `A + D vs. control`—is excluded, thus distributing its total contribution equally as 1/3 per comparison. This example illustrates how contributions are reallocated based on the minimal sets of comparisons essential for maintaining identifiability in sparse networks.

Validation is unnecessary for this theoretical network as it solely demonstrates predefined redistribution rules for unidentifiable components. The contribution values are direct assignments from these rules—not statistically estimated outcomes.

### Real-world dataset utility and validation

**Fig. 3:**
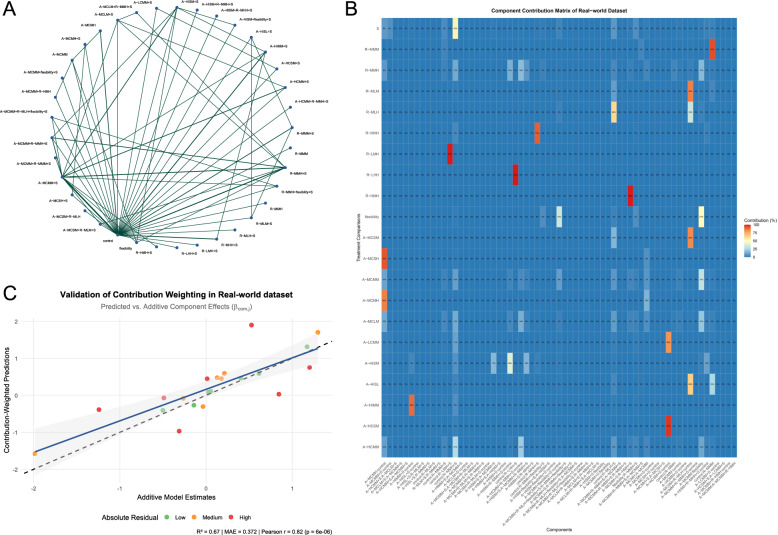
**a** Network plot of real dataset. Unpublished dataset evaluating the efficacy of different exercise modalities on lean mass proportion change in adults with obesity, there are 21 distinct exercise components, 40 multi-component interventions, and 66 direct comparisons in this network. **b** Contribution matrix using leave-one-out method (transposed). Columns represent components; rows denote comparisons; values indicate proportional contributions of each comparison to component effect precision. **c** Validation of contribution-weighted predictions. Scatterplot shows moderate congruence between additive model estimates ($\:{\beta\:}_{\text{com}}$) and contribution-weighted predictions ($\:{\beta\:}_{\text{pred}}$). Dashed line = identity reference; blue band = 95% CI of regression slope. Key metrics: (1) R² (0.67): variance explained; (2) MAE (0.372): average prediction error; (3) Pearson r (0.82): linear correlation strength. Residual colors denote absolute difference tertiles: low (≤ 0.12, green), medium (0.12–0.24, orange), high (> 0.24, red)

The analysis utilized an unpublished CNMA dataset developed by our team, evaluating exercise modalities’ efficacy on lean mass proportion in adults with obesity. Components are abbreviated using initial-letter codes representing distinct exercise types. The network comprised 21 exercise components, 40 multi-component interventions, and 66 direct comparisons (Fig. 3a). Using automated R scripts developed by our team, we computed the contribution matrix to quantify the statistical leverage of comparisons on component effects. For readability, the matrix was transposed (Fig. 3b).

Notably, Table 1 provides the parameter decomposition of direct versus additive evidence using algorithms in Eq. 3 to Eq. 8, while the contribution matrix quantifies precision leverage. Remarkably, these distinct methodological approaches yielded highly concordant results for key components. For R-MMM, the direct comparison’s contribution proportion was 94% (matrix row 2, the 5th column from the right), closely aligning with its 90% direct evidence weight in parameter decomposition. Similarly for A-MCMH, both methods agreed on 84% of contribution from direct evidence in leave-one-out contribution (matrix row 14, column 1) and 84.3% in decomposition. Conversely, A-MCMM showed minimal influence in both frameworks (2.3% contribution matrix [row 13, 11th column from right]; 0.9% decomposition), reflecting its large variance (95%CI: −4.0 to 4.8) and negligible precision impact. Similarly, R-MMH exhibited minimal influence (3.2% contribution matrix [row 3, 12th column from right]; 0.3% decomposition) due to large variance (95%CI: −7.8 to 6.8). This empirical consistency confirms our method’s reliability in identifying key evidentiary anchors. All other components lacked direct evidence, relying 100% on additive evidence.

**Table 1:** Decomposition of component effects into direct and additive evidence sources

Component	Direct estimate with 95%CI ($\:{\varvec{\beta\:}}_{\varvec{d}\varvec{i}\varvec{r}}$)	Direct proportion $\:\left({\varvec{\varphi\:}}_{\varvec{d}\varvec{i}\varvec{r}}\right)$	Additive estimate with 95%CI ($\:{\varvec{\beta\:}}_{\varvec{a}\varvec{d}\varvec{d}}$)	Additive proportion $\:\left({\varvec{\varphi\:}}_{\varvec{a}\varvec{d}\varvec{d}}\right)$	Component estimate with 95%CI ($\:{\varvec{\beta\:}}_{\varvec{c}\varvec{o}\varvec{m}}$)
A-HCMM	NA	0.0%	0.0 (−0.6 to 0.6)	100.0%	0.0 (−0.6 to 0.6)
A-HCSM	NA	0.0%	−0.1 (−2.8 to 2.5)	100.0%	−0.1 (−2.8 to 2.5)
A-HIMM	NA	0.0%	0.1 (−0.9 to 1.1)	100.0%	0.1 (−0.9 to 1.1)
A-HISL	NA	0.0%	−0.3 (−4.4 to 3.8)	100.0%	−0.3 (−4.4 to 3.8)
A-HISM	NA	0.0%	0.2 (−1.1 to 1.4)	100.0%	0.2 (−1.1 to 1.4)
A-LCMM	NA	0.0%	−0.0 (−3.5 to 3.4)	100.0%	−0.0 (−3.5 to 3.4)
A-MCLM	NA	0.0%	−0.5 (−1.1 to 0.1)	100.0%	−0.5 (−1.1 to 0.1)
A-MCMH	−0.4 (−1.8 to 1.0)	84.3%	−0.6 (−2.9 to 1.7)	15.7%	−0.5 (−1.7 to 0.7)
A-MCMM	0.4 (−4.0 to 4.8)	0.9%	−0.3 (−0.7 to 0.2)	99.1%	−0.3 (−0.7 to 0.2)
A-MCSH	NA	0.0%	−1.2 (−4.5 to 2.0)	100.0%	−1.2 (−4.5 to 2.0)
A-MCSM	NA	0.0%	0.6 (−2.3 to 3.5)	100.0%	0.6 (−2.3 to 3.5)
flexibility	NA	0.0%	0.1 (−0.9 to 1.0)	100.0%	0.1 (−0.9 to 1.0)
R-HMH	NA	0.0%	0.5 (−3.1 to 4.1)	100.0%	0.5 (−3.1 to 4.1)
R-LHH	NA	0.0%	1.3 (0.1 to 2.5)	100.0%	1.3 (0.1 to 2.5)
R-LMH	NA	0.0%	−2.0 (−6.5 to 2.5)	100.0%	−2.0 (−6.5 to 2.5)
R-MHH	NA	0.0%	−0.0 (−1.1 to 1.0)	100.0%	−0.0 (−1.1 to 1.0)
R-MLH	NA	0.0%	−0.2 (−2.4 to 2.0)	100.0%	−0.2 (−2.4 to 2.0)
R-MLM	NA	0.0%	−1.2 (−4.1 to 1.7)	100.0%	−1.2 (−4.1 to 1.7)
R-MMH	0.5 (−7.8 to 6.8)	0.3%	0.7 (0.3 to 1.1)	99.7%	0.8 (0.4 to 1.2)
R-MMM	1.3 (0.4 to 2.3)	90.0%	11.1 (8.3 to 14.0)	10.0%	1.2 (0.3 to 2.1)
S	NA	0.0%	0.4 (−0.2 to 1.0)	100.0%	0.4 (−0.2 to 1.0)

For each component, the table presents direct estimates (with 95% CI) when available, direct evidence proportion, additive evidence estimates, additive evidence proportion, and final composite component estimates. NA indicates unavailable direct evidence

We further evaluated the congruence between contribution-weighted predictions and additive model estimates through three metrics: Pearson correlation (*r* = 0.818, *p* < 0.001), explained variance (R² = 0.669), and mean absolute error (MAE = 0.372) (Fig. 3c). Again, this validation serves not as confirmation of mathematical equivalence, but as a diagnostic tool to assess conceptual alignment. The moderate correlation and residual error confirm that while precision-based contributions correlate with effect directionality, they do not function as direct weighting factors in effect size determination. This distinction is particularly evident given heterogeneous comparison variances in the real-world data, contrasting with the artificial equal-variance scenario where stronger numerical agreement was observed in the hypothetical network 1.

## Discussion

The leave-one-out algorithm presented in this study represents a significant advancement in quantifying evidence contributions within the CNMA. It extends the conceptual framework of jackknife-based influence diagnostics to the unique complexities of component-level inference by leveraging variance dynamics, thereby circumventing a core limitation of pathway-based methods: the intractability of path enumeration in CNMA due to component dependencies. Specifically, the precision leverage metric$\:\:{m}_{i,j}\:$quantifies how the exclusion of a direct comparison inflates the variance of a component effect. This captures the comparison’s role in stabilizing the *identifiability* of that component effect, elegantly addressing the challenge of disentangling contributions to individual components when treatments share overlapping elements—a scenario where inherent dependencies confound the graph-theoretic evidence path tracing (e.g., the non-identifiable latent path implied by “A + B vs. B + C”).

The theoretical positioning of this algorithm should be examined within the methodological genealogy of contribution analysis. Contribution quantification in NMA is characterized by two major paradigms: The “variance importance” paradigm emphasizes the stabilizing effect of evidence sources on estimation precision, with its core metric directly reflecting the relative variance inflation caused by the exclusion of studies, without requiring summation to 100% and rejecting the interpretation of percentage contributions; On the other hand, the “flow contribution” paradigm achieves percentage allocation of evidence flow through the deconstruction of the Hat matrix, pursuing the intuitiveness of a total weight sum of 100%. The precision leverage $\:{m}_{i,j}\:$in this study firmly aligns with the former. Its foundation in variance inflation adapts Rücker et al.‘s concept of treatment-level “importance” to the hierarchical structure of CNMA, where treatment effects are linear combinations of latent components. By directly quantifying component-level variance fluctuations caused by excluding comparisons, $\:{m}_{i,j}$serves as a robust probe into the model’s complexity, circumventing the intractable need to decompose unobservable component paths and their entangled evidence flows.

The special rules of contribution redistribution under unidentifiability embody a profound statistical stance: in evidence synthesis, the guarantee of identifiability (finite variance) should take precedence over the allocation of contribution proportions. As demonstrated by component D in Network 1, when a single comparison carries all identifiability, its 100% contribution assignment is mathematically inevitable; while in Network 2, the uniform allocation rule of the minimal identifying set is essentially a compromise to the scarcity of information when evidence sources exhibit complete collinear dependence. The purpose of contribution metrics should be to identify “evidence anchors” rather than enforce percentage allocation.

The mathematical decomposition of component effects into direct and additive evidence sources (Eqs. 3–8) establishes a rigorous foundation for understanding evidence synthesis in CNMA. This framework precisely quantifies how statistical precision is synthesized from distinct evidence streams by leveraging inverse-variance relationships. The direct evidence proportion $\:{\varphi\:}_{\text{dir},j}=\frac{{V}_{\text{com},j}}{{V}_{\text{dir},j}}$ (Eq. 8) measures the relative precision gain when direct evidence is augmented by additive evidence. Empirical validation confirmed this framework’s power: Components with high direct evidence proportions (e.g., R-MMM: 90%, A-MCMH: 84.3%) exhibited near-perfect alignment with their dominance in the contribution matrix (94% and 84%, respectively). This convergence demonstrates that both precision leverage and variance decomposition reliably pinpoint pivotal direct evidence sources that anchor the estimate. Conversely, components like A-MCMM ($\:{\varphi\:}_{dir}$=0.9%) showed negligible contributions from direct evidence in both frameworks, highlighting their inherent dependence on network-derived, model-dependent inference. This synergy is transformative for evidence interpretation: It explicitly quantifies the “evidential pedigree” of component effects, guiding clinicians and guideline developers to discern whether an estimate is empirically grounded (high direct evidence proportion), warranting higher confidence for decision-making, or model-inferred (high additive evidence proportion), requiring greater caution due to reliance on the additivity assumption and network connectivity.

A key insight, corroborated by both our hypothetical and real-data analyses, is the fundamental distinction between precision leverage and effect size weighting. While the congruence test in the equal-variance hypothetical Network 1 showed high correlation (R²=0.99) between contribution-weighted predictions ($\:{\beta\:}_{\text{pred}}$) and model-derived estimates ($\:{\beta\:}_{\text{com}}$), this alignment was significantly weaker in the real-world data with heterogeneous variances (R²=0.67). This divergence explicitly demonstrates that the normalized contributions $\:{M}_{i,j}$ quantify the impact on the statistical precision (variance) of the component estimate, not its role as a linear weight for determining the effect size magnitude in the additive model. This distinction resonates with Rücker et al.‘s emphasis that their importance metric does not represent percentage contributions summing to 100% and should not be misinterpreted as direct weights for effect estimation. Our findings reinforce that precision leverage and pathway-based evidence flow contributions represent distinct conceptual dimensions of “contribution” within the CNMA framework.

### Limitations

While the proposed framework addresses critical gaps in the CNMA, several limitations warrant consideration:

First, the leave-one-out approach, though avoiding path enumeration, requires iterative recalculation of covariance matrices after excluding each comparison. For instance, a large network analyzing 30 components across 200 trials would necessitate 60,000 iterative model fittings, each with a complexity of 30^3^. While our research team has developed automation tools to mitigate practical barriers, scalability remains constrained by hardware resources.

Second, when component identifiability is lost due to comparison deletion, contribution weights are redistributed uniformly or proportionally among dependent components. These rules, though empirically justified, lack a rigorous mathematical foundation and rely on heuristic assumptions. Uniform redistribution assumes equal information loss across collinear components—an assumption contradicted by real-world scenarios where certain components may dominate biological pathways. Future methodologies should formalize redistribution through component-specific prior distributions.

Lastly, the illustrative examples address common scenarios but fail to encompass all potential complexities. In networks with intricate interdependencies (e.g., cyclic component interactions), contribution analysis may require ad hoc adjustments. Such cases resist generalization into a unified mathematical framework, necessitating expert judgment and situation-specific adaptations. This limitation underscores the need for case-by-case validation in atypical evidence networks.

## Conclusions

The leave-one-out algorithm resolves a critical gap in CNMA methodology by providing a robust, variance-based framework for quantifying the contribution of direct comparisons to component effect estimates. It reliably identifies pivotal evidence sources essential for component identifiability and precision across diverse network structures, enhancing the transparency and interpretability of evidence synthesis for complex interventions.

## Data Availability

The datasets and R code used for analysis in this study are available upon reasonable request from the corresponding author.
